# Factors associated with advanced diagnosis of cervical cancer: a hospital-based retrospective study in a state of the Brazilian Legal Amazon

**DOI:** 10.3332/ecancer.2025.2045

**Published:** 2025-11-24

**Authors:** Marco Aurélio Bertúlio das Neves, Noemi Dreyer Galvão, Fernanda Cristina da Silva de Lima, Júlio Fernando Pinto Oliveria, Sancho Pedro Xavier, Ageo Mário Cândido da Silva

**Affiliations:** 1 State Secretary of Health of Mato Grosso, Cuiabá, Mato Grosso 78050-970, Brazil; 2 Institute of Collective Health, Federal University of Mato Grosso, Av Fernando Correa da Costa, nº 2367 - Bairro Boa Esperança, Cuiabá, MT 78060-900, Brazil; 3 Rio de Janeiro Cancer Foundation, Rio de Janeiro, RJ 40226-946, Brazil; 4 National Institute of Cancer (INCA), Rio de Janeiro, RJ 20231-130, Brazil; 5 School of Public Health, University of São Paulo (USP), São Paulo 05508-010, Brazil; a https://orcid.org/0000-0002-0685-9233; b https://orcid.org/0000-0002-8337-0669; c https://orcid.org/0000-0002-7815-4304; d https://orcid.org/0000-0002-9187-527X; e https://orcid.org/0000-0001-9493-4098; f https://orcid.org/0000-0001-5293-9413

**Keywords:** cervical neoplasms, hospital records, health services accessibility, epidemiology, delayed diagnosis

## Abstract

**Background:**

Cervical cancer (CC) is a public health issue and one of the leading causes of morbidity and mortality among women. In Brazil, despite prevention and screening strategies, many cases are still diagnosed at advanced stages. This study aimed to analyse factors associated with advanced CC diagnosis in the state of Mato Grosso between 2002 and 2021.

**Method:**

This is a retrospective study based on data from the Hospital Cancer Registry. A total of 1,126 women diagnosed with invasive CC (ICD-10: C53) were included. Sociodemographic, clinical and treatment access variables were analysed. Multinomial logistic regression was used to assess associations between variables and the stage at diagnosis.

**Results:**

The results showed that 58.3% of women were diagnosed at advanced stages (III and IV). Most patients were between 35 and 59 years old, had incomplete primary education and were non-white. Squamous cell carcinoma was the predominant histological type (71.8%). Women with adenocarcinoma had a lower chance of being diagnosed at an advanced stage, while the probability of a localised diagnosis decreased with age.

**Conclusion:**

The high number of late CC diagnoses suggests barriers to access to screening and early treatment in Mato Grosso. Expanding screening coverage, strengthening human papillomavirus vaccination and improving oncology services are essential to reduce the incidence and mortality of the disease in the state.

**Trial registration:**

Identification/approval number by the Committee of Ethics in Research with Human Beings in the Health Area – CEP of the Federal University of Mato Grosso – UFMT, opinion number: 4.858.521.

## Background

Cervical cancer (CC) is a major global public health challenge, significantly impacting morbidity and mortality in women. It develops due to persistent damage caused by infection with oncogenic subtypes of the human papillomavirus (HPV), particularly HPV-16 and -18, which are responsible for the majority of cases [1, 2]. Globally, CC is estimated to be the fourth most common cancer among women [3]. In Brazil, excluding non-melanoma skin tumours, it is the third most incident primary location and the fourth most common cancer mortality in women [1].

In Brazil, an estimated 17,010 new cases of CC are expected annually for the period 2023–2025, corresponding to a crude incidence rate of 15.38 cases per 100,000 women. In 2023, CC is projected to be the third most common cancer among women in the Midwest region (16.66/100,000), the second in the Northeast (17.59/100,000) and North (20.48/100,000), the fourth in the South (14.55/100,000) and the fifth in the Southeast (12.93/100,000). Among the states in the Midwest region, CC is estimated to rank fourth in Mato Grosso (12.33/100,000), second in Mato Grosso do Sul (21.77/100,000) and Goiás (17.47/100,000) and third in the Federal District (14.47/100,000) [1].

The World Health Organisation and the National Cancer Prevention and Control Policy within the Unified Health System (SUS) (Law 14.758 of December 19, 2023) recommend prevention actions, early detection and timely access to treatment [4, 5].

Despite these recommendations and the availability of the vaccine against HPV, more than half of new diagnoses are of an advanced stage [6]. In Brazil, this proportion was 31.5% in 2001, and in 2019, it was 32.4% and the mortality rate adjusted by the world population was 4.79 deaths/100,000 women in 2022, the highest recorded since 2005. In the Midwest region, it was the third leading cause of cancer death in women, with a mortality rate of 5.05/100,000 women. Mato Grosso had the highest rate among the states in this region, 5.54 deaths/100,000 women [7]. These rates are usually higher in less developed countries, since this tumour is closely related to poor living and health conditions [8].

Several factors contribute to delayed diagnosis, particularly in low-resource settings, where barriers such as limited access to screening programs, low adherence to preventive measures and disparities in healthcare infrastructure persist [9, 10].

In Brazil, late diagnosis of CC remains a major challenge, particularly in regions with geographical and structural barriers to healthcare access, such as Mato Grosso.

Analysis of the risk factors associated with squamous cell carcinoma and cervical adenocarcinoma reveals significant differences that have implications for prevention and treatment. Understanding these associations is fundamental to developing screening and intervention strategies that can reduce the incidence and mortality of CC.

Considering this perspective and that knowledge of the sociodemographic, epidemiological and clinical characteristics associated with the diagnosis of advanced-stage CC are essentials for planning actions in both primary care and highly complex oncology units, as well as for improving indicators of timely diagnosis and treatment, this study adopted as its guiding question: what is the temporal evolution of CC cases diagnosed at an advanced stage, and what are the associated factors in the reality of Mato Grosso? In this context, the objective of the study was to analyse cases of advanced-stage CC and associated factors in the state of Mato Grosso.

## Methods

This is a retrospective study that evaluated the association of sociodemographic and clinical characteristics with the advanced stage of women who have had CC in the state of Mato Grosso.

All the information on health units (clinics and hospitals that provide cancer care) in Mato Grosso was obtained from the Hospital Cancer Registry Integrator (HCR), available on the National Cancer Institute website (https://irhc.inca.gov.br/RHCNet), accessed on December 19, 2023 [11].

The study population consisted of women diagnosed with invasive CC (code C53 of the 10th Revision of the International Statistical Classification of Diseases and Related Health Problems – ICD-10) reported by the HCR between 2002 and 2021, with histopathological confirmation, age at the first visit calculated by the difference between the date of the first visit and the date of birth and with available information on staging.

Only patients classified by the HCR as analytical (cases for which the main treatment was carried out at the institution responsible for the record) were included.

The cases in which the Federative Unit (UF) of treatment was different from the patient’s place of birth were considered in the analysis in order to approximate a hypothetical situation of a barrier to access.

All cases of *in situ* or intraepithelial neoplasms, as well as benign or non-invasive tumours, were excluded. In addition to sarcomas, lymphomas and neuroendocrine tumours, as these are entities whose occurrence, diagnosis, risk factors and treatment are different from those related to the pathology that is the subject of this study.

The variables analysed were:

- schooling (none; incomplete primary education; complete primary education; secondary education; incomplete higher education; complete higher education);
- federation unit of birth (person’s UF of birth);
- race/skin color (white and non-white (black, yellow, brown, indigenous and yellow));
- marital status (married/stable union, separated, single and widowed);
- age (in age groups, the first of which was made up of women under 30; the other categories were defined by intervals of every 5 years, up to 70 or more years);
- previous diagnosis and treatment (without diagnosis, without treatment; with diagnosis, without treatment; with diagnosis, with treatment; Other);
- histological type (Epidermoid; Adenocarcinoma; Other – including tumours that do not fit into the previous categories);
- Completeness of treatment (Completed treatment; did not complete treatment);
- family history of cancer (yes; no);
- smoking (never smoked; former smoker; yes - current smoker);
- alcoholism (never used alcohol; former alcoholic; yes - current alcoholic);
- stage at diagnosis: According to the International Federation of Gynecology and Obstetrics classification for staging, stage I CC is that which is restricted to the uterus. Stage II tumours are those that have spread beyond the uterus without, however, reaching the pelvic bones or the lower third of the vagina. Stage III tumours are those that affect the pelvic wall, the lower third of the vagina, or cause ureteral obstruction. Stage IV includes tumours that invade the mucosa of the rectum or bladder, or that spread at a distance. The cases were classified according to their stage at the time of diagnosis. Thus, group 1 (initial disease) included those with stage I; group 2 (localised disease) included those with stage II; and group 3 (advanced disease) included those with stages III and IV.
- Health Region (Upper Tapajós, Baixada Cuiabana, Araguaia Xingu, Northern Araguaia Karajá, Center North, Garças Araguaia, Middle Araguaia, Middle North, North, Northwest, West, Southwest, South, Teles Pires, Arinos Valley, Peixoto Valley).

The study outcome stage at diagnosis was used as the response variable, where cases were dichotomised into I. as localised disease and advanced disease, and II. initial disease, this category being chosen as the reference category.

Initially, a descriptive analysis of all the study variables was carried out, followed by a crude analysis of the selected variables. Finally, the multinomial logistic regression model and the Wald test were applied to check the significance of individual coefficients or a set of coefficients in the model. The variables selected for the multiple models were those with an association with the outcome of *p*-value ≤0.20 and which had a maximum of 35% of records with no information. The crude and adjusted odds ratios (OR) were calculated, along with their respective 95%CI. Statistical analyses were carried out using R *software* version 4.3.1 (R Core Team, 2023).

With regard to ethical aspects, the data made available by the HCR Integrator, the cancer cases, are in the public domain and have unrestricted access (https://irhc.inca.gov.br/RHC-Net). Therefore, it was not necessary for the study protocol to be examined by the Human Research Ethics Committee, as recommended by the National Health Council.

## Results

The study analysed 1,126 women who met the inclusion criteria. Women aged 45 to 49 accounted for 14.2% of the cases, 61.9% were concentrated in the 35 to 59 age group and 17.1% had incomplete primary education (Table 1).

In relation to the location to the place where the women were treated, 48.8% were born in the same place as the treatment in Mato Grosso, 20.3% had never smoked and 23.1% had never drunk. The proportion of women with no information about smoking and drinking were 68.0% and 69.4%, respectively. In terms of marital status, 33.7% of the women were separated, 61.5% were non-white and 57.6% of the cases were from the more developed health regions (Baixada Cuiabana and South) (Table 1).

Regarding clinical characteristics and those related to the diagnosis and treatment of women diagnosed with CC, 92.8% of the women completed the initial treatment proposed and 13.9% had stable disease at the end of it, although this variable contained 58.1% of the observations with no information. Most of the women (79.0%) arrived at the referral institution with a firm diagnosis (Table 2).

With regard to the family history of cancer, 16.8% had no history of cancer. Regarding the histological type of cancer, 71.8% were epidermoid. The percentage of stages III and IV diagnoses at the time of diagnosis was 58.3% of cases (Table 2).

**Figure 1.**
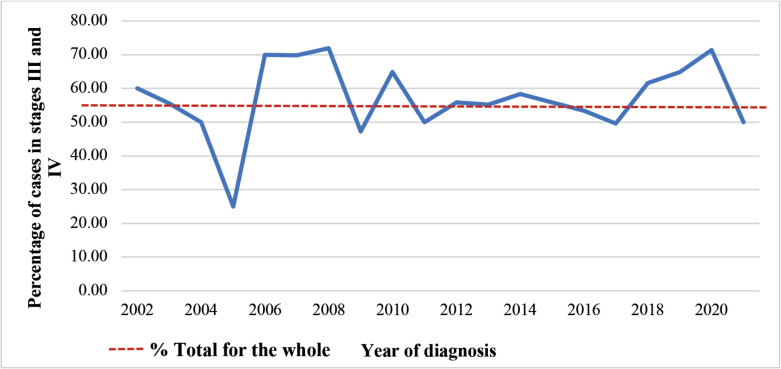
Temporal evolution of the percentage of cases of women diagnosed with stages III and IV CC, according to HCR, Mato Grosso, 2002–2021.

Figure 1 shows the temporal evolution of the percentage of cases of women diagnosed with stages III and IV CC, according to HCR in Mato Grosso from 2002 to 2021. The data reveal significant fluctuations in the percentage of cases over the study period. In 2005, the percentage was the lowest recorded (25.0%), while in subsequent years, it remained consistently above 50.0%.

In the crude analysis, the chance of being diagnosed with the disease in the localised categories decreased with increasing age, with the difference becoming statistically significant from the 30–39 age group onwards and between the 45–59 age group. Compared to women aged up to 30, women aged 30 to 59 have a 33.7% to 21.7% lower chance of having localised disease Table 3.

The chance of having advanced disease diagnosed with adenocarcinoma histological type was lower (OR = 0.86; 95%CI: 0.78;0.94) than in women with epidermoid histological type Table 3.

When comparing women living in the Baixada Cuiabana Health Region, women living in the West Health Region were more likely to have localised disease (OR = 1.36; 95%CI: 1.07;1.74), as well as advanced disease (OR = 1.20; 95%CI:1.04;1.38). Women living in the Centro Norte Health Region had lower chances of developing localised disease (OR = 0.76; 95%CI: 0.59;0.96). In the Teles Pires Health Region, on the other hand, they were more likely to develop advanced disease (OR = 1.12; 95%CI: 1.01; 1.25) Table 3.

There was no statistically significant association between the following variables: state of birth and treatment, race/skin color and marital status.

The adjusted model consisted of all the variables included in the crude analysis. In this model, the chance of having a diagnosis of the disease in the localised categories decreased with increasing age, with the difference becoming statistically significant from the 30–39 age group and between the 45–59 age group. Compared to women with epidermoid histological type, women with adenocarcinomas were less likely to have advanced disease (OR = 0.53; 95%CI: 0.33; 0.88) Table 3.

Women who underwent treatment in a UF other than the one where they were born, compared to those who underwent treatment in the same UF where they were born, non-white women compared to white women and separated, single and widowed women compared to married women, showed no significant difference for any of the disease categories (localised and advanced) Table 3.

When comparing women living in the Baixada Cuiabana Health Region, women living in the West Health Region were more likely to develop localised disease (OR = 5.92; 95%CI: 1.54;39.27), as well as advanced disease (OR = 5.54; 95%CI:1.61; 34.92). Meanwhile, those living in the Teles Pires Health Region were more likely to develop advanced disease (OR = 2.60; 95%CI: 1.22; 6.25) Table 3.

## Discussion

This study examined the evolution of cases of women diagnosed with CC in the state of Mato Grosso from 2002 to 2021, with a focus on the associated factors. The analysis revealed that more than half of the cases in Mato Grosso were diagnosed at an advanced stage of CC. Furthermore, the study found that in 85.0% of the years in the historical series analysed, the percentages found were equal to or greater than 50.0%. The predominant histological type was epidermoid, accounting for 71.7% of cases. The highest percentages occurred in women aged between 45 and 49, with incomplete primary education, who had never smoked or drunk, who were separated, non-white women, without a family history of cancer, who arrived at the treatment unit with a definite diagnosis and with stable disease after completion of the initially proposed treatment. The majority of cases resided in the Baixada Cuiabana and South health regions and the most common histologic type was epidermoid. The probability of being diagnosed with CC in the localised category decreased with increasing age of the women and the diagnosis of advanced disease with histological type adenocarcinoma was lower than in women with epidermoid type.

Among the health regions that exhibited statistically significant outcomes, a notable disparity emerged in the prevalence of localised disease among female populations. Specifically, women residing in the Center North health region demonstrated a 24% higher incidence of localised disease compared to their counterparts in the Baixada Cuiabana health region. In contrast, individuals residing in the West health region exhibited an increased propensity for both localised and advanced diseases. A similar trend was observed in the Teles Pires Health Region, where residents were also more susceptible to advanced disease.

The finding in this study that the diagnosis of advanced disease with the histological type adenocarcinoma was lower than in women with the epidermoid type was consistent with the findings of several studies carried out in Brazil and the UK [12, 13]. However, this study contradicts the prevailing idea in the literature that adenocarcinomas would be identified at more advanced stages than squamous tumours due to their location (endocervical canal) and the fact that symptoms are less evident compared to epidermoid tumours [14, 15].

The elevated proportions of advanced-stage diagnoses documented in this study suggest that the cancer care policy in Mato Grosso has been unsuccessful in leveraging a pivotal element in enhancing the probability of cure, adopting less aggressive treatment modalities and enhancing the quality of life for patients. This phenomenon is not unique to Mato Grosso; it is also observed in Brazil. In Goiás, the percentage of advanced-stage diagnoses was 47.7% between 2013 and 2020 [16], in Rio de Janeiro, it was 67.7% between 2012 and 2014 [17] and in Bahia, just over 38.0% between 2008 and 2017 [18].

A cross-sectional study conducted in two specialised cancer hospitals in Nepal revealed that 80.0% of CC cases were diagnosed late and were more likely to occur in non-literate women who delayed in sharing their symptoms [19]. Population-based cross-sectional studies carried out in Ethiopia and Ghana found more than 2/3 of cases with advanced diagnosis [20]. This phenomenon stands in stark contrast to the evolution and severity of the disease, thereby presenting a significant challenge to health systems in developing countries as they strive to ensure the population has unimpeded access to early detection and timely treatment [18].

This trend may be indicative of the profound social inequalities and the ineffectiveness of public policies in addressing them. Given the inherent association between public policies and the social policies implemented by the state, it is anticipated that health policies are influenced by numerous determinants and conflicting interests, ultimately shaping their direction and format [21]. Perhaps the theoretical perspectives analysing the relationship between the state and the development of social policies, which argue that the state serves the interests of the ruling class and perpetuates social and economic inequalities to the detriment of the needs of the majority, is a possibility to be investigated [22].

The undesired evolution in the prevalence of diagnoses at an advanced stage of CC can be explained, together with other factors, such as social, demographic, cultural and health characteristics, access to health services, biological characteristics of the disease and lack of screening. Revealing a complex interrelationship between these factors.

Analysis of the results of different studies reveals that, while squamous cell carcinoma is more prevalent in populations with a history of HPV infection (types 16 and 18) [23, 24], while adenocarcinoma may be more associated with factors such as age, hormonal conditions and exposure to estrogens interacting with HPV infection in the development of this type of CC [25].

The occurrence of the latter has been recorded in younger women, especially under the age of 25. The rationale behind the increased prevalence of cervical adenocarcinoma in young women is attributable to the higher frequency of HPV types associated with adenocarcinoma in this demographic, including HPV-18 and -45 [26, 27]. This observation suggests that the demographic profile and risk factors associated with adenocarcinoma may influence detection and diagnosis at more advanced stages.

Although this study did not find an association between age and diagnosis at an advanced stage of CC, a study carried out in Brazil that analysed women with CC at the time of diagnosis using the HCR integrator between 2000 and 2012 [6] found a positive association between older women and a diagnosis of CC in more advanced stages.

Despite the controversy in the literature regarding the effect of age on the staging of CC, evidence suggests that lower adherence to screening, procrastination by women in seeking these tests, a possibly shorter pre-invasive period and changes due to the senescence of the immune system may be related to the risk of diagnosis at an advanced stage among the older population [12].

Although the education variable was not included in the analysis of association with diagnosis at an advanced stage, given the high percentage of ‘no information’ in the database used, educational level and socioeconomic status cannot be disregarded. A correlation between lower educational attainment and women from lower socioeconomic backgrounds with a higher risk of CC has been documented [18, 28, 29], which is likely attributable to diminished awareness of and access to preventive health services [29, 30].

This study also found no statistical significance for race/skin color; however, a time series analysis of mortality from CC according to race/skin color in Brazil from 2002 to 2021 found that black women die more and have a lower drop in the coefficient, also finding an increase in racial inequality over the years [31]. A study conducted in Florida also demonstrated that factors such as socioeconomic status, race and ethnicity significantly influence the stage at which CC is diagnosed, with African-American and Hispanic women having higher rates of diagnosis at advanced stages. This phenomenon can be attributed to inequalities in access, healthcare and health education [32]. These disparities are often exacerbated by structural factors, such as geographical location and the availability of adequate health services.

A cross-sectional study conducted in Saudi Arabia, 2022, highlighted the critical role of health beliefs in the use of screening. It revealed a worryingly low uptake of CC screening (24.6%), despite a significant willingness (45%) to participate, indicating a gap between intention and action that may be influenced by individual characteristics and perceived barriers by users [33]. In this sense, understanding individual risk factors is important for improving prevention strategies.

The perception and recognition of CC symptoms play a crucial role in early diagnosis. Many women do not recognise the importance of early symptoms and, even when they do seek care, may face delays in diagnosis due to the inability of health professionals to quickly identify the signs of the disease. This is exacerbated in regions where health education is limited and where women may not have adequate information about the prevention and signs of CC [34]. Health education is therefore an essential strategy for reducing diagnoses at advanced stages.

A systematic review on CC in Morocco emphasised that the implementation of screening programs and the promotion of HPV vaccination were essential to reduce the incidence of this cancer in advanced stages. The lack of effective public health programs and low adherence to HPV vaccination are factors that contribute to the increase in this reality in many developing countries [35].

The finding in this study that almost 71.0% of the women arrived at the treatment unit with a confirmed diagnosis possibly points to an improvement in the effectiveness of the actions of the less complex health care units in identifying diagnostic suspicions and diagnosis.

Among the limitations of this study, it is important to highlight the use of secondary data, as well as the restriction of information made available by the HCR and other health information systems that record cases of the disease. It is well known that the use of this information leads to a greater possibility of under-registration, under-reporting, errors in filling in data, coding and the incompleteness found in some variables. In addition to making it impossible to include them in the analysis, they may have had an impact on the interpretation of the results and part of the effect found in the trends in the percentages of advanced diagnosis may be due to these factors. In this sense, it could be hypothesised that under-registration primarily affects more advanced cases. Thus, the percentage of diagnoses at an advanced stage could be even higher than that found in this study.

Another limitation worth highlighting is the fact that the HCR covers SUS users, failing to record almost all people treated by the supplementary health system. If the distribution of the outcome among users of this system is significantly different from that observed in the sample studied, this may constitute a selection bias. In the SUS, the data comes from a population of public health services. Due to the predominance of people from lower socioeconomic classes, they have a higher prevalence of certain chronic diseases, less access to cutting-edge technologies and specialised tests, as well as possible barriers to accessing quality health care, such as long queues and travel difficulties. On the other hand, data from private clinics tends to represent patients with a higher socioeconomic status, who have better financial conditions and often faster access to health services. This group may have a different epidemiological profile, with fewer cases of diseases linked to poverty, access to more modern treatments and a higher frequency of preventive visits to the doctor.

Even considering the progressive improvement in coverage recorded in the HCR in Mato Grosso, this study found a situation similar to that of another study using the same database [6], in that the total number of cases recorded was much lower than the estimated annual incidence of the disease. This fact reinforces the need to make progress in improving the quality and feeding of the Hospital Registry in Mato Grosso in a more consistent and systematic way.

On the other hand, it is important to highlight the positive points of this study, the identification of health regions and population subgroups for which educational and health actions aimed at the early detection of CC in the state should be prioritised.

## Conclusion

The trend of advanced-stage CC diagnoses found in this study shows that the health system in Mato Grosso faces a major challenge in guaranteeing the population full access to early diagnosis and timely treatment. The findings confirmed that socio-economic disparities are associated with advanced-stage CC, as well as showing that users face problems in health services that prevent them from accessing a referral health unit in a timely manner. There is a need to expand access to cancer treatment services in the state of Mato Grosso, especially for these groups that had a higher prevalence of advanced-stage CC diagnoses, as well as the quality of data in the RHC needs to be improved and cancer control policies in Mato Grosso can take into account differences in access, the actions and services provided and the characteristics of health regions to improve their efficiency.

## Conflicts of interest

The authors declare that there are no competing interests.

## Funding

No funding was received for the conduct of this study.

## Author contributions

The conception and design of the study were developed by Marco Aurélio Bertúlio das Neves, Noemi Dreyer Galvão and Ageo Mário Candido da Silva. The preparation of the material, data analysis and interpretation were carried out by Marco Aurélio Bertúlio das Neves, Noemi Dreyer Galvão, Fernanda Cristina da Silva de Lima e Ageo Mário Candido da Silva. The first draft of the manuscript was written by Marco Aurélio Bertúlio das Neves and all other authors contributed to the critical revision of the content and subsequent versions of the manuscript and approved the final manuscript, namely: Marco Aurélio Bertúlio das Neves, Noemi Dreyer Galvão, Ageo Mário Candido da Silva, Fernanda Cristina da Silva de Lima, Júlio Fernando Pinto Oliveira and Sancho Pedro Xavier.

## Figures and Tables

**Table 1.** Sociodemographic characteristics of women diagnosed with CC who met the inclusion criteria for the study, according to HCR, Mato Grosso, 2002–2021.

Sociodemographic characteristics	*n*	%
Age		
<30	59	5.2
30–34	107	9.5
35–39	128	11.4
40–44	148	13.1
45–49	160	14.2
50–54	148	13.1
55–59	113	10.0
60–64	82	7.3
65–69	76	6.7
≥70	105	9.3
Education		
None	73	6.5
Incomplete elementary school	193	17.1
Complete elementary school	138	12.3
High school	79	7.0
Higher education incomplete	5	0.4
Higher education complete	31	2.8
No information	607	53.9
Federation unit of birth		
Same as UF of treatment	550	48.8
Differs from UF of treatment	522	46.4
No information	54	4.8
Smoking		
Never	229	20.3
Former smoker	52	4.6
Yes	76	6.7
Not evaluated/Not applicable	3	0.3
No information	766	68.0
Alcoholism		
Never	260	23.1
Former alcoholic	24	2.1
Yes	55	4.9
Not evaluated/Not applicable	5	0.4
No information	782	69.4
Marital status		
Married	332	29.5
Separated	380	33.7
Single	75	6.7
Widow	37	3.3
No information	302	26.8
Race/skin color		
White	232	20.6
Not white	693	61.5
No information	201	17.9
Health region		
Middle Araguaia	21	1.9
Upper Tapajós	35	3.1
Baixada Cuiabana	434	38.5
Garças Araguaia	51	4.5
West	53	4.7
North	20	1.8
Center North	36	3.2
Arinos Valley	15	1.3
Northwest	26	2.3
Peixoto Valley	22	2.0
Southwest	24	2.1
Araguaia Xingu	19	1.7
South	215	19.1
Northern Araguaia Karajá	3	0.3
Teles Pires	87	7.7
Middle North	65	5.8

Source: HCR Integrator

**Table 2.** Clinical characteristics and characteristics related to diagnosis and treatment of women diagnosed with CC who met the inclusion criteria for the study, Mato Grosso, 2002–2021.

Clinical characteristics and those related to diagnosis and treatment	*n*	%
Treatment at the institution		
Completed treatment	1,045	92.8
Did not complete treatment	64	5.7
No information	17	1.5
Disease status at the end		
No evidence of disease	74	6.6
Partial remission	39	3.5
Stable disease	157	13.9
Progressing disease	89	7.9
Out of therapeutic possibility	21	1.9
Death	65	5.8
Not applicable	27	2.4
No information	654	58.1
Family history of cancer		
Yes	141	12.5
No	189	16.8
No information	796	70.7
Previous diagnosis and treatment		
No diagnosis, no treatment	230	20.4
With diagnosis, without treatment	796	70.7
Diagnosed, with treatment	93	8.3
Other	6	0.5
No information	1	0.1
Histologic type		
Epidermoid	808	71.8
Adenocarcinomas	125	11.1
Others	193	17.1
Stage of diagnosis		
I	189	16.8
II	280	24.9
III	489	43.4
IV	168	14.9

Source: HCR integrator

**Table 3.** Association between sociodemographic and clinical characteristics of women with CC and localised and advanced staging of the disease, Mato Grosso, 2002–2021.

Demographic and clinical characteristics	Localised disease		Advanced disease	
	OR _crude_ (95% CI)	OR _adjusted_ (95% CI)	OR _crude_ (95% CI)	OR _adjusted_ (95% CI)
Age				
< 30	1	1	1	1
30–34	0.66 (0.53; 0.83)	0.14 (0.04; 0.44)	0.86 (0.3; 1.02)	0.49 (0.15; 1.37)
35–39	0.74 (0.59; 0.92)	0.19 (0.05; 0.57)	0.91 (0.78; 1.07)	0.57 (0.17; 1.62)
40–44	0.86 (0.7; 1.07)	0.38 (0.11; 1.17)	0.97 (0.83; 1.14)	0.84 (0.25; 2.41)
45–49	0.76 (0.62; 0.94)	0.27 (0.08; 0.79)	0.92 (0.78; 1.07)	0.62 (0.19; 1.72)
50–54	0.79 (0.64; 0.97)	0.27 (0.08; 0.81)	0.91 (0.78; 1.07)	0.56 (0.17; 1.57)
55–59	0.78 (0.63; 0.97)	0.28 (0.08; 0.87)	0.9 (0.76; 1.06)	0.64 (0.19; 1.86)
60–64	0.8 (0.63; 1.01)	0.29 (0.08; 0.98)	0.92 (0.77; 1.09)	0.57 (0.16; 1.79)
65–69	0.77 (0.6; 1.00)	0.32 (0.08; 1.14)	0.97 (0.81; 1.15)	0.98 (0.27; 3.18)
≥70	0.84 (0.67; 1.06)	0.36 (0.09; 1.2)	0.97 (0.82; 1.15)	0.95 (0.27; 2.94)
Federation unit of birth				
Same as UF of treatment	1	1	1	1
Differs from UF of treatment	1.05 (0.96; 1.15)	1.19 (0.78; 1.85)	0.99 (0.94; 1.05)	0.91 (0.62; 1.32)
Histological type				
Epidermoid	1	1	1	1
Adenocarcinomas	0.89 (0.79; 1.02)	0.73 (0.41; 1.31)	0.86 (0.78; 0.94)	0.53 (0.33; 0.88)
Others	1.01 (0.9; 1.14)	1.16 (0.67; 2.04)	0.97 (0.9; 1.05)	0.87 (0.55; 1.39)
Race/skin color				
White	1	1	1	1
Not White	1.02 (0.9; 1.14)	1 (0.57; 1.73)	1.01 (0.94; 1.09)	1.08 (0.69; 1.68)
Marital status				
Married	1	1	1	1
Separated	1.03 (0.91; 1.16)	1.13 (0.66; 1.95)	1.01 (0.95; 1.09)	1.17 (0.76; 1.82)
Single	1.02 (0.84; 1.25)	1.05 (0.43; 2.65)	0.99 (0.88; 1.12)	0.78 (0.37; 1.74)
Widow	0.98 (0.75; 1.24)	0.74 (0.25; 2.28)	0.92 (0.78; 1.08)	0.49 (0.2; 1.28)
Health region				
Baixada Cuiabana	1	1	1	1
Middle Araguaia	1.00 (0.77; 1.30)	0.99 (0.31; 3.32)	0.80 (0.63; 1.00)	0.42 (0.13; 1.38)
Upper Tapajós	1.21 (0.95; 1.54)	2.19 (0.69; 8.42)	1.05 (0.88; 1.26)	1.81 (0.62; 6.66)
Garças Araguaia	1.13 (0.92; 1.38)	1.6 (0.63; 4.39)	0.99 (0.85; 1.15)	1.29 (0.55; 3.31)
West	1.36 (1.07; 1.74)	5.92 (1.54; 39.27)	1.20 (1.04; 1.38)	5.54 (1.61; 34.92)
North	1.05 (0.75; 1.49)	0.94 (0.21; 4.96)	1.03 (0.83; 1.28)	1.45 (0.42; 6.75)
North Center	0.76 (0.59; 0.96)	0.26 (0.07; 0.79)	0.86 (0.74; 1.00)	0.48 (0.22; 1.08)
Arinos Valley	0.84 (0.55; 1.3)	0.46 (0.58; 3.04)	1.00 (0.80; 1.26)	1.29 (0.36; 6.11)
Northwest	1.14 (0.83; 1.55)	1.6 (0.39; 8.28)	1.09 (0.90; 1.31)	2.27 (0.7; 20.23)
Peixoto Valley	1.10 (0.79; 1.52)	1.2 (0.28; 6.21)	1.05 (0.85; 1.29)	1.23 (0.37; 5.60)
Southwest	0.81 (0.6; 1.09)	0.41 (0.1; 1.48)	0.89 (0.74; 1.07)	0.67 (0.25; 1.90)
Araguaia Xingu	0.88 (0.64; 1.22)	0.61 (0.14; 2.5)	0.90 (0.73; 1.12)	0.80 (0.26; 2.79)
South	1.05 (0.93; 1.18)	1.14 (0.66; 1.99)	0.99 (0.92; 1.07)	1.25 (0.77; 2.04)
Northern Araguaia Karajá	1.53 (0.59; 3.98)	*	1.26 (0.71; 2.24)	*
Teles Pires	1.16 (0.96; 1.41)	2.05 (0.85; 5.37)	1.12 (1.01; 1.25)	2.60 (1.22; 6.25)
Middle North	0.86 (0.68; 1.08)	0.64 (0.23; 1.72)	1.04 (0.93; 1.17)	1.42 (0.71; 3.09)

* Could not be adjusted as *n* is small
